# Myocardial Mechanics and Associated Valvular and Vascular Abnormalities in Left Ventricular Noncompaction Cardiomyopathy

**DOI:** 10.3390/jcm13010078

**Published:** 2023-12-22

**Authors:** Attila Nemes

**Affiliations:** Department of Medicine, Albert Szent-Györgyi Medical School, University of Szeged, 6725 Szeged, Hungary; nemes.attila@med.u-szeged.hu; Tel.: +36-62-545220; Fax: +36-62-544568

**Keywords:** left ventricular, non-compaction, cardiomyopathy, cardiac mechanics, vascular, remodeling

## Abstract

Left ventricular (LV) non-compaction (LVNC) is a rare genetic cardiomyopathy due to abnormal intra-uterine arrest of compaction of the myocardial fibers during endomyocardial embryogenesis. Due to the partial or complete absence of LV compaction, the structure of the LV wall shows characteristic abnormalities, including a thin compacted epicardium and a thick non-compacted endocardium with prominent trabeculations and deep intertrabecular recesses. LVNC is frequently associated with chronic heart failure, life-threatening ventricular arrhythmias, and systemic embolic events. According to recent findings, in the presence of LVNC, dysfunctional LV proved to be associated with left atrial volumetric and functional abnormalities and consequential dilated and functionally impaired mitral annulus, partly explaining the higher prevalence of regurgitation. Although the non-compaction process morphologically affects only the LV, signs of remodeling of the right heart were also detected. Moreover, dilation and stiffening of the aorta were present. The aim of the present detailed review was to summarize findings regarding changes in cardiac mechanics, valvular abnormalities, and vascular remodeling detected in patients with LVNC.

## 1. Noncompaction Cardiomyopathy

Left ventricular (LV) noncompaction (NC) cardiomyopathy (NCCM) or spongiform cardiomyopathy is a rare disorder due to abnormal intra-uterine arrest of compaction of the myocardial fibers during endomyocardial embryogenesis between the fifth and eighth weeks of gestation [1,2,3,4]. In a significant number of LVNC cases, mutations of genes encoding sarcomeric or cytoskeletal proteins have been shown, but an acquired case of peripartum evolution of the acquired LVNC syndrome has also been demonstrated [5]. The pathology was first detected by Engberding and Bender in 1984, but at that time it was called ventricular sinusoidosis based on the echocardiographic images seen [2]. Later, after clarifying the origin of the disease, the term “LV noncompaction” (LVNC) began to be used. Normally, LV compaction occurs from the epicardium to the endocardium and from the basal part of the LV to the apex. These facts explain why we find abnormalities on the endocardial side in patients with NCCM and why the LV apical segment is almost always affected. Due to these abnormalities, characteristic complications of NCCM include chronic heart failure, life-threatening ventricular arrhythmias, and systemic embolic events [2,3,4]. The prevalence of NCCM in patients referred to echocardiography laboratories ranges between 0.014 and 1.26% [5].

Due to the partial or complete absence of LV compaction, the structure of the LV wall shows characteristic abnormalities, including a thin compacted epicardium and a thick non-compacted endocardium with prominent trabeculations and deep intertrabecular recesses. Due to the rarity of the disease, currently only limited data are available on myocardial mechanics, valvular abnormalities, and vascular remodeling in a larger group of NCCM patients. Therefore, the present paper aimed to summarize these LVNC-associated abnormalities in a series of patients. Literature data of isolated RV [6] or biventricular [7] forms of NCCM were not included in this review. Case reports, rare coincidences, and associations of LVNC and other disorders, such as congenital, atherosclerotic, secondary valvular, or other diseases, are not included in this paper. Arrhythmologic and thromboembolic consequences of LVNC were also not analyzed. There is a special group of patients in whom LVNC and coronary artery disease are present at the same time, and it is not known whether regional wall motion abnormalities are caused by the coronary artery disease or the noncompaction of the myocardium in most cases [8].

## 2. Cardiovascular Imaging and Criteria

**Figure 1:**
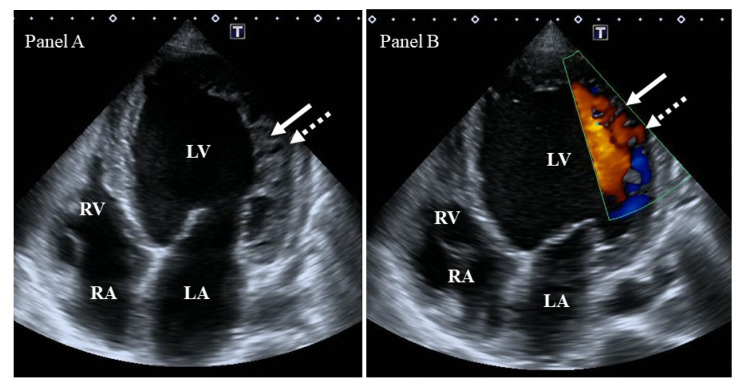
Two-dimensional grey-scale image (**Panel A**) and Doppler evidence (**Panel B**) of left ventricular (LV) noncompaction demonstrating LV lateral wall trabeculation (white arrows) and sinuses (dashed white arrows). Abbreviations: LV = left ventricle, LA = left atrium, RV = right ventricle, RA = right atrium.

During the last few years, cardiac computed tomography and cardiac magnetic resonance imaging (cMRI) have become an integral part of routine clinical practice due to the advances in imaging and computing software and hardware. In addition, echocardiography has developed significantly, and new modalities have appeared and become part of the everyday practice. As a result, more detailed volumetric and functional chamber quantifications, analysis of myocardial mechanics and valvular morphology and function, and more detailed assessments of vascular function have become clinically available. For the diagnosis of NCCM, several echocardiographic and cMRI-derived criteria are used [9]. The most important ones are detailed below:

Echocardiographic criteria (Figure 1):

- Chin’s criterion is the earliest and simplest criterion: The distance between the epicardial surface and the trough of the trabeculation divided by the distance between the epicardial surface and the peak of the trabeculum measured at the apex of the LV in parasternal short-axis and apical views is ≤0.5 [3].
- Jenni’s criterion is more complicated and takes into account a number of other factors: (I) the presence of a two-layer myocardial structure with a thin compacted and a thicker noncompacted myocardium, (II) the ratio of noncompacted to compacted myocardium > 2 at the end-systole, (III) the absence of coexisting cardiac structural abnormalities, and (IV) excessive prominent trabeculations and deep intertrabecular recesses filled with intraventricular blood with color Doppler imaging. Parameters are measured in parasternal short-axis views [4].
- Stollberger’s criterion is a simplified criterion that is closer to the clinical routine: (I) the presence of >3 trabeculations located apically to the papillary muscles in parasternal short-axis and apical views, within a distinct two-layered myocardium in the end-systole, and (II) perfusion of intertrabecular recesses with either color Doppler or echocardiographic contrast imaging in the end-diastole [10].

cMRI criteria:

- Petersen’s criterion is simple, as it aims to assess the noncompacted/compacted myocardial ratio, ideally with a ratio > 2.3 in the end-diastole, excluding the LV apex from the measurement, as the compacted myocardium is physiologically thinner [11].
- Jacquier’s criterion aims to assess trabecular LV mass, and a value > 20% of global LV mass in the end-diastole is diagnostic [12].
- Grothoff’s criterion is more complicated: (I) LV myocardial mass index of the noncompacted tissue (‘a’) >15 g/m^2^, (II) ‘a’ as a percentage of total LV myocardial mass index >25%, and (III) increased trabeculation in LV basal segments and a noncompacted/compacted ratio ≥3:1 measured in the end-diastole on the short axis [13].

In addition to the routine methods used, imaging procedures such as three-dimensional (3D) speckle-tracking echocardiography (3DSTE) have become available [14,15,16,17]. Three-dimensional STE uses virtual 3D models with which 3D volume measurements of all chambers can be performed simultaneously with assessment of deformation (strain) and rotational mechanics using the same acquired 3D echocardiographic datasets. In this review, findings were summarized regarding patients with LVNC who participated in the Motion Analysis of the heart and Great vessels bY three-dimensionAl speckle-tRacking echocardiography in Pathological cases (MAGYAR-Path) study, which started in 2011 and was conducted in the Department of Cardiology at the University of Szeged, Hungary. The MAGYAR-Path study was designed to assess the clinical efficacy and the prognostic value of 3DSTE-derived parameters of patients with various pathologies including NCCM [18,19,20,21,22].

The present review aimed to summarize findings related to LVNC in myocardial, valvular, and vascular structural and functional properties regardless of which imaging method was used, including ones published in the frame of the MAGYAR-Path study. Of the vascular parameters, only ones related to the aorta and pulmonary artery were detailed.

## 3. The Left Heart and the Aorta

### 3.1. Left Ventricle

#### 3.1.1. Under Healthy Circumstances

**Figure 2:**
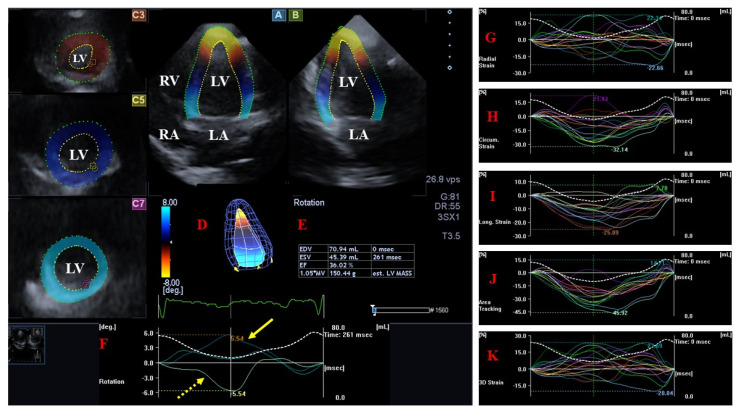
Evaluation of the left ventricle by three-dimensional (3D) speckle−tracking echocardiography. Following the acquisition of a 3D echocardiographic dataset and by using a dedicated vendor-provided software, several views are produced automatically: longitudinal apical four-chamber (**A**) and two-chamber (**B**) views and short-axis views at the apical (**C3**), midventricular (**C5**), and basal (**C7**) LV regions. The 3D LV model (**D**), calculated LV volumes and ejection fraction (**E**), and apical (yellow arrow) and basal (yellow dashed arrow) LV rotations (**F**), together with time–LV global (white curve) and segmental (colored curves) radial (**G**), circumferential (**H**), longitudinal (**I**), area (**J**), and 3D (**K**) strain curves with a time–LV volume change curve (dashed white curve), are demonstrated. The image presents a patient with reduced LV function (decreased LV ejection function) and normally directed LV rotational mechanics with reduced counterclockwise apical (yellow arrow) and preserved clockwise basal (dashed yellow arrow) LV rotations. Abbreviations: LV = left ventricle, LA = left atrium, RV = right ventricle, RA = right atrium, EDV = LV end-diastolic volume, ESV = LV end-systolic volume, EF = LV ejection fraction, MASS = LV muscle mass.

The LV is an egg- or bullet-shaped cardiac chamber. Muscle bands and papillary muscles limit obtaining the optimal contours in order to enable accurate measurements. In healthy subjects, during systole, the mitral valve (MV) closes and the aortic valve (AV) opens, allowing blood flow from the LV to the aorta, whereas during diastole, blood flows into the LV from the left atrium (LA) across the open MV. During this phase of the cardiac cycle, the AV is closed. In the LV, there are subepicardial fibers running in a left-handed direction, fibers in the mid-layer running circumferentially, and subendocardial fibers running in a right-handed direction [23,24]. Movements of the LV wall follow a 3D pattern, including radial thinning/thickening, longitudinal lengthening/shortening, and circumferential widening/narrowing during the cardiac cycle. This myocardial deformation in 3D can be represented by echocardiographic strains: simple unidimensional/unidirectional radial (LV-RS), longitudinal (LV-LS), and circumferential (LV-CS) strains, with the combination of them being area strain (LV-AS, combination of LV-LS and LV-CS) or 3D strain (LV-3DS, combination of all strains) [14,15,16,17,23]. Furthermore, these movements of the LV wall are not independent; the basal regions of the LV rotate in a clockwise direction during systole, whereas the apex of the LV moves in the opposite counterclockwise direction under healthy circumstances. These opposite basal and apical rotations result in an LV twist, which leads to the LV wringing during systole. In diastole, LV untwisting is seen, during which the basal and apical LV regions move in opposite directions compared to the movements during systole [23,24,25] (Figure 2).

#### 3.1.2. In Noncompaction Cardiomyopathy

##### LV Structure, Volumes, and Function

In an early study, the extension of NC myocardium was predominantly seen at the apex (72%), with an LV ejection fraction (EF) < 50% in 83% of the patients. Hypokinesis was observed in both noncompacted and compacted segments [26]. In another pool of LVNC patients, 60% of the patients were in New York Heart Association (NYHA) functional class III/IV and 79% had systolic dysfunction (LV-EF < 50%). A dilated left heart with systolic dysfunction was found to be one of the predictors of an adverse outcome [27]. In another pool of isolated sub-Saharan African LVNC patients, 63% of the subjects had NYHA functional class II, and heart failure due to systolic dysfunction was the most common clinical presentation (98%). The LV end-diastolic diameter was dilated and LV-EF was impaired. Common sites of noncompaction were the apical (100%), midinferior (74.1%), and midlateral (64.8%) regions [28].

Wall motion abnormalities were present in the majority of the compacted LV segments in LVNC patients [29,30,31]. The wall motion score (WMS) index was markedly abnormal in the compacted LV segments of LVNC patients but significantly less abnormal compared to the noncompacted segments [32]. In a recent real-time 3D echocardiographic study, contributions of compacted and noncompacted segments to global LV systolic dysfunction were examined in LVNC. Noncompacted and compacted LV segments had comparable increased regional volumes and reduced systolic function, suggesting that systolic LV dysfunction observed in LVNC is not confined to noncompacted LV segments [32]. There was an inverse correlation between the noncompacted area and LV-EF, suggesting that noncompaction itself contributes to LV dysfunction [33]. In contrast, paradoxical contributions of noncompacted vs. compacted segments to global LV dysfunction were found in another study: Relatively less impairment in the WMS index in case of the noncompacted segments was seen compared to the compacted value of the LV segments [34]. Moreover, a strong correlation was found between systolic and diastolic dysfunction in LVNC by tissue Doppler imaging [35]. No relationship between LV radial wall motion and longitudinal velocity and the extent and severity of NCCM could be demonstrated [36].

##### LV Strains

Compared with controls, patients with LVNC with preserved LV-EF had similar LV systolic function and chamber dimensions but a larger mass and greater relative wall thickness, more abnormal LV geometry, and decreased global LV-LS [37]. In other studies, global LV-CS and LV-RS proved to be reduced in the same population [38,39]. Although LV trabecular muscle mass was very different between men and women, no disease-related strain differences were found between men and women [38]. In a further study, patients with LVNC with normal LV-EF showed impaired global LV-RS, LV-CS, and LV-LS. The extent of excessive trabeculation was higher in LVNC compared to controls, and this value positively correlated with LV end-diastolic and end-systolic volume indices, and stroke volume index but negatively correlated with global LV-RS and LV-CS [40].

It was also demonstrated in LVNC patients that global LV-LS was reduced regardless of LV-EF [41,42] and that global LV-LS and QRS duration were associated with LV-EF in LVNC [43]. Global and regional LV-LS and LV-CS were impaired in LVNC, and apical peak LV-CS was strongly associated with cardiovascular events [44]. The myocardial strain of the cardiac apex and endocardium was significantly lower than that of the cardiac base and epicardium, respectively. Myocardial strain reduction was more significant in LVNC patients with focal myocardial fibrosis [45]. LVNC patients with heart failure (HF) with preserved LV-EF have diffuse fibrosis, which is more extensive at the apical level, explaining the decrease in apical deformation. Lower transmural and base-to-apex deformation gradients support the sequence of myocardial maturation failure [46]. The strain values changed as LV-EF decreased, but the LVNC-specific LV strain pattern could not be detected [47]. In LVNC patients with severe HF, LV-EF increased, but LV volumes did not change during the follow-up after cardiac resynchronization therapy. A total of 33% of patients proved to be respondent, with a reduction in inter-ventricular, longitudinal, and radial intra-ventricular dyssynchrony [48]. The presence of myocardial ischemia in NCCM patients is associated with LV dilation and worse LV function represented by LV-EF and global LV-LS [49].

All 3DSTE-derived strains proved to be significantly decreased in all segments of LVNC patients compared to segments of controls. However, LV-RS and LV-3DS showed further reduction in noncompacted segments compared to compacted segments (results from the MAGYAR-Path Study) [22]. This was partly confirmed when severely diminished myocardial efficiency was found in LVNC, and LV function seemed to depend mainly on the compact myocardial wall layer [50]. The impairment of all strains correlated well with the extent of the non-compacted myocardium as well [51]. In contrast, lower regional deformation values in compacted segments compared to noncompacted segments with relatively preserved apical deformation compared to basal segments were found in another study on LVNC [52].

Beyond the above fact, a significant decrease in LV-EF, fractional shortening, E/E’, and global LV strains could be detected in the relatives of the patients compared to healthy volunteers [53]. Moreover, carriers of multiple genetic variants had a lower LV-EF and cardiac index, increased LV fibrosis, and reduced global LV-CS in LVNC [54].

In addition to studies conducted in adults [55], extensive studies have also been carried out in children. LVNC in pediatric patients was associated with LV enlargement and impaired LV systolic function, represented by lower LV-EF and LV strains [56]. In another study, LV myocardial deformation was found to be decreased in longitudinal and circumferential dimensions by 2DSTE in NCCM children [57]. In LVNC children with normal LV-EF, LV-LS and LV-CS values were reduced [58]. In a number of studies, impairment of LV-EF, end-diastolic dimension, or LV posterior wall compaction and decreased LV strains were associated with adverse outcomes in children [59,60]. Global and segmental LV-RS, LV-CS, and LV-LS were decreased in pediatric patients with LVNC [56,61], and the lowest segmental values were found in those with adverse outcomes compared with those with benign outcomes [61].

Naturally, the possible use of LV strains in differential diagnosis was also raised. Global LV-LS showed significant differences between LVNC and dilated cardiomyopathy (DCM) [62,63]. The base–apex longitudinal gradient of the strain appears as a valuable additive tool for distinguishing LVNC from DCM [63]. Regarding the early findings, a special regional deformation pattern with preserved deformation in basal segments of LVNC can help to differentiate LVNC from DCM as well [64]. In contrast, LV volumes and global LV-LS and LV-CS were similar in DCM and in LVNC, but the trabeculated and papillary muscle index was higher and apical LV-CS was significantly lower in LVNC than in DCM. These minor alterations might be due to the morphological characteristics of LVNC with a trabeculated apical region [65]. In another study, LV segmental strain analysis revealed that basal LV-CS was lower in DCM than in LVNC. In correspondence with previous findings, both median and apical LV-LS were lower in LVNC than in DCM; moreover, apical LS was the most effective in distinguishing LVNC from DCM [66]. In an early study examining the differentiation of LVNC and hypertrophic cardiomyopathy (HCM), an increased number of trabeculations, a thinner maximal wall thickness, and a lower LV-EF with homogeneously reduced myocardial function were found in LVNC, whereas an apical-to-basal gradient with relatively preserved apical function was present in HCM [67]. LVNC showed a more significant reduction in LV-LS in the apical region compared to patients with HCM, suggesting an abnormality of development in these regions [68]. 

LVNC patients with mitral valve (MV) regurgitation had more severe morphological and functional LV changes [69]. In another study, LVNC patients with MV regurgitation showed significant deterioration in LV myocardial strains and impaired LV geometry and function, including lower LV-EF and greater LV end-systolic volume and LV mass compared to LVNC patients without MV regurgitation. Moreover, the incidence of adverse outcomes may be related to the degree of MV regurgitation in these cases [70].

##### LV Rotational Mechanics

In a recent study, LV rotational parameters of healthy adolescent athletes meeting Jenni’s criteria for LVNC were not worse compared to those who did not meet the criteria [71]. LV twist was found to be significantly reduced in young patients with LVNC compared with controls and those with LV hypertrabeculation, and reduced LV twist was an independent predictor of LVNC [72]. In contrast, the peak LV twist values were comparable between LVNC patients and controls [37]. In another study, the rotation of basal LV segments was reduced and the rotation of apical LV segments was much lower, resulting in reduced LV twist [44,73]. Apical LV rotation and net LV twist were lower in NCCM/LVNC patients compared to controls [42,51,53], but both basal and apical LV rotations were reduced in patients with LVNC with LV-EF < 50% [41,42]. The impairment of LV twist parameters correlates well with the extent of the non-compacted myocardium [51]. Upon comparing patients and their relatives, despite no difference being found in the patients’ relatives, a decreasing pattern in rotation values was found [53]. LV twist was associated with cardiovascular events [44]. There was no difference in the rotational pattern between DCM and LVNC, and both healthy and patient populations showed heterogeneous rotational patterns [65].

##### LV Rigid Body Rotation

In some cases, both basal and apical LV segments move in the same direction, resulting in an absence of LV twist. NCCM/LVNC is the most examined disorder in which a near absence of LV twist, known as LV rigid body rotation (LV-RBR), was found in 26% to 100% of cases [37,41,47,53,68,72,73,74,75,76,77]. It was suggested early on that LV-RBR may be a new sensitive and specific, objective and quantitative, functional diagnostic criterion for NCCM [74]. LV-RBR had 88% specificity and 78% sensitivity in differentiating NCCM from LV hypertrabeculation [75]. Similar results were found in children, in whom LV-RBR was present in 56% of NCCM patients and in 4% of subjects with hypertrabeculation [72]. LV-RBR was present in 100% [74], 88% [75], 57% [41], 56% [72], 53% [53,73], 50% [68], 39% [76], and 26% of cases [44] in a series of NCCM/LVNC patients. LVNC patients with LV-RBR showed worse NYHA functional status but similar signs of remodeling, LV-EF, and strain values compared to those with LVNC and normally directed LV rotational mechanics [73]. The presence and direction of LV-RBR seemed to be related to LV-EF: Patients with lower LV-EF (<50%) had higher rates of clockwise LV-RBR (39%), whereas subjects with LV-EF > 50% had a ratio of 26% of counterclockwise LV-RBR [47]. In another study, the ratio of LV-RBR proved to be 92.3% vs. 26.7% in the same subgroups, respectively [41]. Patients with reverse apical rotation (clockwise LV-RBR) had lower LV-LS but similar LV-EF compared with patients without reverse apical rotation [76]. In most cases, LV-RBR proved to be clockwise oriented in NCCM [53,72,73,74,75,76]. When NCCM patients and their first-degree relatives were compared, 53% and 30% showed LV-RBR with the same ratio of a dominantly clockwise vs. counterclockwise pattern, respectively [53]. The prevalence of LV-RBR was significantly increased (57% vs. 14%, *p* = 0.05) in the LVNC population compared to controls [37].

### 3.2. Left Atrium

#### 3.2.1. Under Healthy Circumstances

**Figure 3:**
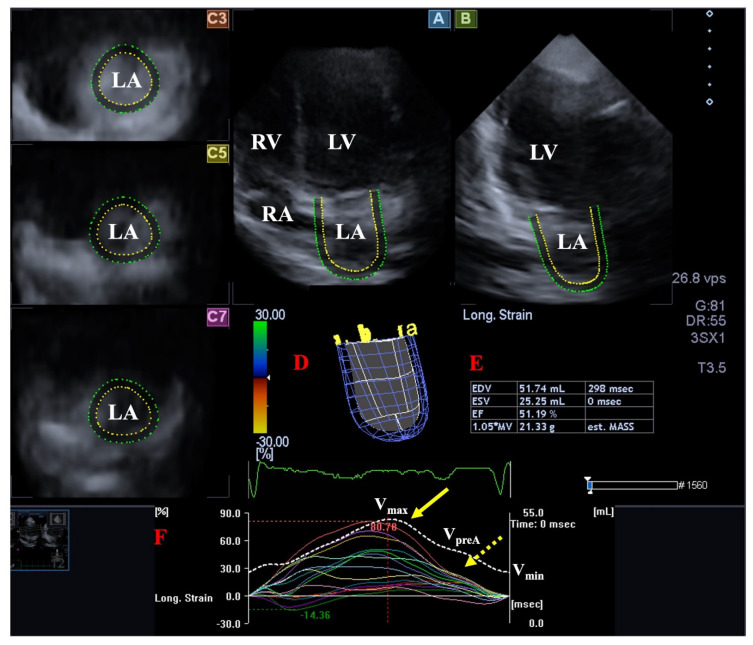
Evaluation of the left atrium (LA) by three-dimensional (3D) speckle-tracking echocardiography. Following acquisition of a 3D echocardiographic dataset and by using a dedicated vendor-provided software, several views are produced automatically: Longitudinal apical four-chamber (**A**) and two-chamber (**B**) views, together with short-axis views at the basal (**C3**), midatrial (**C5**), and superior (**C7**) LA regions, are demonstrated. A virtual 3D LA cast (**D**), together with calculated LA volumes (**E**) and time–LA global (white curve) and segmental (colored curves) longitudinal (**F**) strain curves with a time–LA volume change curve (dashed white curve), are also demonstrated. The yellow arrow represents peak LA strains, whereas the yellow dashed arrow represents LA strains at atrial contraction. Abbreviations: LV = left ventricle, LA = left atrium, RV = right ventricle, RA = right atrium, EDV = end-diastolic volume, ESV = end-systolic volume, EF = ejection fraction, MASS = LA muscle mass, Vmax = minimum end-systolic LA volume, VpreA = early diastolic LA volume before atrial contraction, Vmin = end-diastolic minimum LA volume.

The LA has circumferential (e.g., interatrial band, located at the base) and longitudinal (e.g., septoatrial band, located parietally) muscle bands. The rim of the oval fossa has an important role, because the other main muscles of the atrium are attached to it. The LA has a significant role in modulating the filling of the LV. It serves as a reservoir for pulmonary venous flow during LV systole and as a conduit for pulmonary venous return during early LV diastole, as well as a booster pump that enhances ventricular filling during late LV diastole. The volume of the LA is maximal at end-systole just before MV opening and is minimal at end-diastole when the MV closes, and there is a pre-atrial contraction volume directly before atrial systole. Several stroke volumes and emptying fractions could be calculated as functional properties from LA volumes to evaluate all phases of LA function. The Frank–Starling mechanism applies to the LA, meaning that an increase in LA wall contractility can be detected in response to increased LA preload up to a point, beyond which this correlation disappears (Figure 3) [78,79,80].

#### 3.2.2. In Noncompaction Cardiomyopathy

In LVNC, signs of LA remodeling [37] and a larger LA diameter were detected [41,81], which was related to a higher risk of thromboembolism in LVNC patients, suggesting that it may be a useful predictor for thrombotic risk stratification [81]. LA ejection force as a characteristic of LA systolic function was found to be increased in LVNC patients compared to healthy individuals, suggesting a compensation of LA work against the dysfunctional LV [82]. Significantly greater LA volumes, smaller LA emptying fractions, and reduced peak global LA-RS, LA-CS, and LA-AS representing systolic function were present in LVNC patients with reduced LV-EF, as assessed by 3DSTE in the MAGYAR-Path Study [20]. In a recent study, the LA reservoir strain was an independent predictor for high-risk HF events in LVNC patients, and LV-GLS was an independent determinant of the LA reservoir strain [83]. Compared to negative genotype LVNC patients, subjects with a positive genotype had a larger LA volume, a lower LA reservoir, and booster pump strains. Moreover, LA volume can be used to discriminate patients with a positive genotype, as well as those with multiple genetic mutations [54].

### 3.3. Mitral Valve

#### 3.3.1. Under Healthy Circumstances

**Figure 4:**
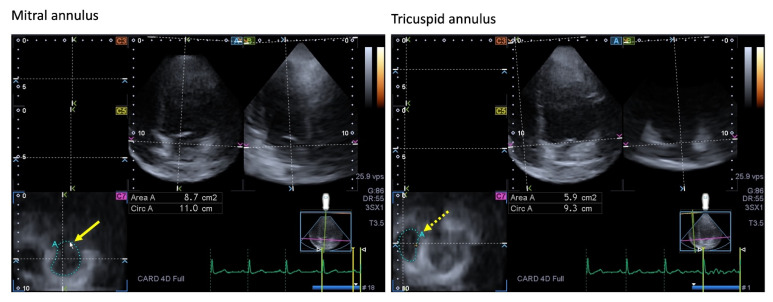
Images showing two-dimensionally projected views of the mitral (MA) and tricuspid (TA) annuli as assessed by three-dimensional (3D) speckle-tracking echocardiography. After the acquisition of the 3D echocardiographic dataset, the following views were produced: Apical four-chamber (**A**) and two-chamber views (**B**) and a cross-sectional view at the level of the MA/TA were optimized in apical four- and two-chamber views (**C7**). The yellow arrow represents the two-dimensional projection of the MA plane, whereas the yellow dashed arrow represents the two-dimensional projection of the TA plane. Abbreviations: LA = left atrium, LV = left ventricle, RA = right atrium, RV = right ventricle, Area = MA/TA area, Circ = MA/TA perimeter.

The MV or left bicuspid atrioventricular valve is a saddle-shaped structure in 3D with a dynamic motion during the cardiac cycle. The MV is composed of the fibrous mitral annulus (MA), the anterior and the posterior leaflets, the tendineal chords, and the papillary muscles. The MV allows normal blood flow from the LA to the LV during diastole and prevents blood backflow (mitral regurgitation) from the LV to the LA during systole. Timely contraction of adjacent LA and LV areas is necessary for proper contraction of MV under healthy circumstances (Figure 4) [84,85,86].

#### 3.3.2. In Noncompaction Cardiomyopathy

MA was dilated and functionally impaired in LVNC patients with impaired LV systolic function with a higher incidence and severity of mitral regurgitation. The number of non-compacted segments did not correlate with MA dimensions and functional properties. No, mild, and moderate to severe MV regurgitation could be detected in 45%, 35%, and 20% of subjects, respectively [87]. In the MAGYAR-Path Study, 33% − 33% of patients showed regurgitation of grades 1 and 2 MV [18]. In other studies, 48.2% and 78% of the subjects showed MV regurgitation [69,88].

### 3.4. Aortic Valve

#### 3.4.1. Under Healthy Circumstances

The aortic valve (AV) separates the LV outflow tract from the ascending aorta and has three semilunar thin leaflets ensuring one-way blood flow towards the systemic circulation. It opens during ventricular systole and closes during ventricular diastole in order to prevent blood flowing from the ascending aorta backwards into the LV [89].

#### 3.4.2. In Noncompaction Cardiomyopathy

In pools of patients with bicuspid AV, the prevalence of associated LVNC proved to be 0.2% [90], 3.4% [91], and 11% [92]. Jenni’s, Petersen’s, and Fazio’s LVNC criteria were associated with BAV in 10.1%, 62.0%, and 54.4% of patients, respectively. These results confirmed that BAV patients do not harbor more LVNC than the general population, and there is no evidence that they are at higher risk for the development of LVNC cardiomyopathy [93]. In a recent study, 19 out of 46 LVNC patients (41%) showed AV regurgitation [88].

### 3.5. Aorta

#### 3.5.1. Under Healthy Circumstances

The aorta is the largest artery carrying blood from the LV to the body. There is a significant interaction between the cardiac chambers and the vasculature. The aorta is an elastic tube and has a significant role in regulating blood flow via the Windkessel effect. Aortic stiffness can be associated with increasing systolic blood pressure (BP) and decreasing diastolic BP, subsequently increasing LV afterload and potentially leading to LV hypertrophy, thereby impairing LV relaxation and potentially causing LV diastolic dysfunction and damaging coronary perfusion [94,95].

#### 3.5.2. In Noncompaction Cardiomyopathy

In a cross-sectional cohort study of 109 NCCM patients, the prevalence of ascending aortic dilatation was 7%, which involved only mild dilatations and was not significantly different from an age- and sex-matched cohort of dilated cardiomyopathy patients [96]. Increased aortic stiffness with no dilation but reduced pulsatile change in aortic diameter was observed in patients with NCCM with moderate to severe HF, confirming previous findings. Changes in aortic stiffness was theorized to be due to HF-induced neurohormonal changes, not to LVNC itself [97].

## 4. The Right Heart and the Pulmonary Artery

### 4.1. Right Ventricle

#### 4.1.1. Under Healthy Circumstances

The right ventricle (RV) is triangular in shape when viewed from the front and curves over the LV. It is crescent shaped in cross-section, with its diameter gradually increasing from the apex to the base. The RV fills from the RA during diastole through the tricuspid valve (TV) and empties into the pulmonary artery through the pulmonary valve (PV) during systole. The muscular wall of the RV, not including trabeculations, is 3–5 mm thick, resulting in a lower muscle mass compared to that of the LV (RV muscle mass is only one-fifth to one-sixth of that of the LV) [98]. The activation sequence of the RV begins in the inlet and the latest activation occurs in the outflow tract. The RV is composed of a free wall containing predominantly transverse fibers subepicardially, with deep subendocardial fibers arranged longitudinally from base to apex. The longitudinal fibers are responsible for the longitudinal shortening, during which the RV axis is shortened and the TV moves in an apical direction. The circumferential fibers play a role in the inward (radial) movement of the RV free wall (“bellows effect”). The superficial muscle fibers of the RV continue onto the LV, contributing to the interdependence of the two ventricles. The RV motion is regulated by the heart rate, the Frank–Starling mechanism, and the autonomic nervous system; the rotation and twisting motions seen in case of the LV do not play an essential role in the RV [99,100,101]. The RV is more trabeculated than the LV even in healthy subjects [102].

#### 4.1.2. In Noncompaction Cardiomyopathy

Analysis of RV morphology in 105 LVNC patients found greater RV apical trabecular thickness among those with an LV end-systolic noncompacted-to-compacted ratio ≥ 2 compared with those with an LV end-systolic noncompacted-to-compacted ratio < 2 or the normal control group. There was no difference between the groups in relation to the RV end-diastolic noncompacted-to-compacted ratio [102]. In another study, RV systolic dysfunction was present in a non-negligible proportion of patients with isolated LVNC; LV systolic function was found to be the only variable independently related to RV systolic function [103]. LVNC was associated with increased trabeculations of the RV apex [102]. In other studies, the RV myocardium displayed more trabeculations in LVNC [104], even is children [55]. However, overlap with normal individuals was extensive, not allowing for the differentiation of patients with LVNC from controls [104]. In a pool of 54 sub-Saharan African LVNC patients, RV noncompaction occurred in 22.2% of patients with RV dilation (74.1%) and depressed RV function (59.3%) [28]. The correlation of LV and RV trabeculation was observed only in LVNC patients with normal LV-EF, whereas LV trabeculation correlated with RV volumes in both types of LV-NC patients regardless of LV-EF. LVNC patients with reduced LV-EF had worse RV strains than patients with LVNC with normal LV-EF, but RV strains correlated with RV trabeculation predominantly in LVNC patients with reduced LV-EF [105]. Subclinical impairment of RV myocardial deformation was present even in children with LVNC [56]. The prevalence of LVNC-related clinical features was similar in patients with RV hypertrabeculation vs. RV normotrabeculation. Patients with an LVNC phenotype might have RV non-compaction with subclinical RV dysfunction and without more severe clinical features [106]. RV dysfunction was present in half of LVNC patients, seems to be a marker of advanced disease [107], and is strongly associated with adverse clinical events and prognosis [102,107,108].

### 4.2. Right Atrium

#### 4.2.1. Under Healthy Circumstances

**Figure 5:**
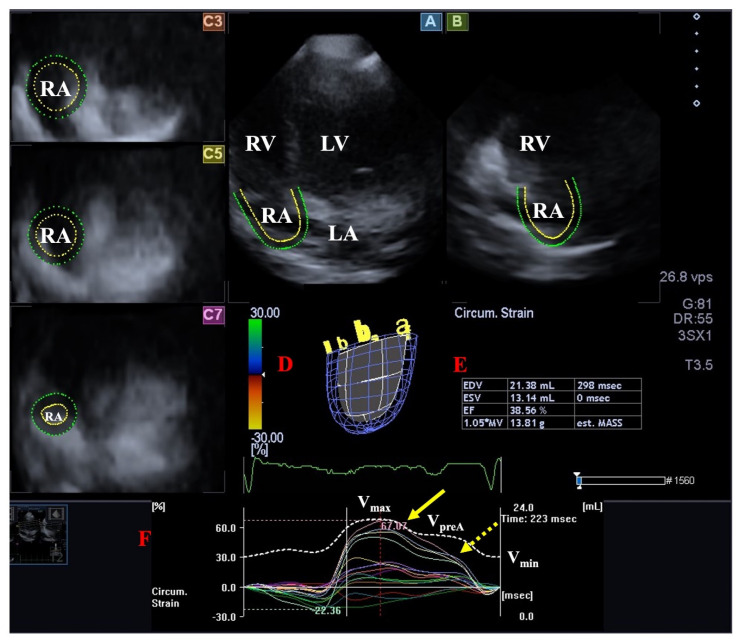
Evaluation of the right atrium (RA) by three-dimensional (3D) speckle-tracking echocardiography. Following acquisition of a 3D echocardiographic dataset and by using dedicated vendor-provided software, several views were produced automatically: Longitudinal apical four-chamber (**A**) and two-chamber (**B**) views and short-axis views at basal (**C3**), midatrial (**C5**), and superior (**C7**) RA levels. The 3D cast of the RA (**D**) is shown with calculated RA volumes (**E**) and time–RA global (white curve) and segmental (colored curves) longitudinal (**F**) RA strain curves together with a time–RA volume change curve (dashed white curve). The yellow arrow represents peak RA strains, whereas the yellow dashed arrow represents RA strains at atrial contraction. Abbreviations: LV = left ventricle, LA = left atrium, RV = right ventricle, RA = right atrium, EDV = end-diastolic volume, ESV = end-systolic volume, EF = ejection fraction, MASS = RA muscle mass, Vmax = minimum end-systolic RA volume, VpreA = early diastolic RA volume before atrial contraction, Vmin = end-diastolic minimum RA volume.

There are circumferential and longitudinal muscular bundles in the RA. The most important muscles are the terminal crest and the terminal pectinate muscles. The rim of the oval fossa has an important role, as the other main muscles of the atrium are attached to it. The RA has a complex structure and serves as a reservoir for the systemic flow return from the caval veins and the coronary sinus during systole, preparing blood to be transferred to the RV and pulmonary circulation. During early diastole, the TV opens and the RV fills passively (RA conduit function), followed by an active RA contraction at the end of diastole (“booster pump” function). Additionally, the sinus node is located in the RA, and the RA plays a role in the production of atrial natriuretic peptides regulated by RA tension and baroreceptors located in the RA wall (Figure 5) [109].

#### 4.2.2. In Noncompaction Cardiomyopathy

Results from the MAGYAR-Path Study found increased cyclic 3DSTE-derived RA volumes in patients with isolated LVNC and reduced LV-EF. However, only mild RA functional alterations, including increased RA stroke volumes in systole and early diastole without RA strain abnormalities, could be detected in LVNC [19].

### 4.3. Tricuspid Valve

#### 4.3.1. Under Healthy Circumstances

The right atrioventricular or tricuspid valve (TV) has a 3D, saddle-shaped, asymmetrical ellipsoid annulus, with a dynamic nature respecting the cardiac cycle, three leaflets, and a subvalvular apparatus [110]. The TV functions as a one-way valve. It opens during ventricular diastole, allowing blood flow from the RA into the RV, and it closes during ventricular systole to prevent the regurgitation of blood from the RV back into the RA (Figure 4).

#### 4.3.2. In Noncompaction Cardiomyopathy

Results from the MAGYAR-Path Study confirmed that the tricuspid annulus (TA) was dilated, with preserved sphincter-like function (TA fractional area change, TAFAC and TA fractional shortening, TAFS) in patients with isolated LVNC and impaired systolic function. Longitudinal (TA plane systolic excursion, TAPSE) and sphincter-like TA movements (TAFAC, TAFS) correlated with each other. Moreover, TA dilation was associated with an increased RA volume with respect to the cardiac cycle. In this study, TV regurgitation grades 1, 2, and 4 were detected in four (27%), one (7%), and one (7%) patients, respectively [18]. In another study, 36 out of 46 LVNC patients (78%) showed TV regurgitation, of which 8 (17%) had moderate/severe TVR [88].

### 4.4. Pulmonary Valve

#### 4.4.1. Under Healthy Circumstances

The pulmonary valve (PV) is a semilunar valve that separates the RV from the pulmonary artery. The PV opens at ventricular systole and closes at ventricular diastole [111].

#### 4.4.2. In Noncompaction Cardiomyopathy

In a recent study, 6 out of 46 LVNC patients (13%) showed PV regurgitation [88].

### 4.5. Pulmonary Artery

#### 4.5.1. Under Healthy Circumstances

The pulmonary artery is a large vessel of the pulmonary system that carries deoxygenated blood from the RV to the lungs being a low-pressure, low-resistance system [101].

#### 4.5.2. In Noncompaction Cardiomyopathy

In a pool of 54 isolated sub-Saharan African LVNC patients, pulmonary hypertension was documented in 83.3% of patients [28]. In another study, 35% of LVNC patients showed pulmonary hypertension [37].

## 5. Pathophysiologic Background

The genetic background of LVNC is both isolated and familial, with autosomal dominant and recessive mitochondrial gene abnormalities and X-linked inheritance [112,113,114,115]. Regarding van Waning et al., 80 genes are thought to have an influence in causing LVNC, including sarcomere genes (e.g., ACTC1, DES, etc.), transcriptional/translational genes (e.g., NKX2–5, NONO etc.), mitchondrial function genes (e.g., NNT, TAZ etc.), cytoskeletal protein genes (e.g., DMD, DTNA, etc.), cellular junction protein genes (DSP, PKP2), intracellular trafficking genes (LAMP2, PLEKHM2), ion channel genes (HCN4, SCN5A), signal transduction genes (ALPK3, DMPK), and a protein degradation gene (MIB1) [113,114,115]. Molecular mechanisms of the specific impaired signaling pathways were established in several studies. Moreover, the inhibition of signaling and genome correction of certain mutations were sufficient as well, suggesting that the application of gene-editing techniques can have a role in the diagnosis and maybe in the treatment of NCCM in the future [116].

Shortly, the alterations detailed above can be traced back to the LV compaction abnormalities underlying the pathology. These abnormalities can lead to various manifestations of heart failure in a significant number of cases. Abnormalities in the LV may explain atrial and valvular morphological and functional alterations, as well as the reason for being more significant on the left side of the heart than on the right side of the heart. In accordance with ventricular–arterial coupling, abnormalities in the heart and large vessels have a mutual effect on each other, explaining vascular abnormalities [1,4].

There are several animal and cell culture models of LVNC that can help identify pathways involved in ventricular noncompaction. Gene-editing technologies using certain enzymes can be used for modeling LVNC. Patient-specific induced pluripotent stem cell-derived cardiomyocytes also proved to be useful for investigating LVNC [117].

## 6. Clinical Implications

LVNC is considered to be a rare pathology with special clinical consequences, including arrhythmologic and thromboembolic complications and heart failure. Although it was first described a little over 30 years ago, today we know quite a lot about the origins, symptomatology, diagnosis, and treatment options of the disease. Due to advances in cardiovascular imaging over the past decades, the diagnosis of LVNC has become easier and we have learned completely new information. The information available today that is outlined and summarized above can help clinicians recognize and hopefully treat the disease more easily. The most important abnormalities are detailed in Table 1.

## 7. Conclusions

LVNC is associated with significant LV volumetric, functional, strain, and rotational abnormalities. There are other LVNC-associated abnormalities of other heart chambers and significant valvular and vascular abnormalities as well.

## Figures and Tables

**Table 1:** Summary of the most important findings.

		Reference Number	Number of LVNC/NCCM pts in the Current Study
**LEFT HEART**			
**Left ventricle**	Hypokinesis was observed in both noncompacted and compacted segments.	[26]	18
	Most patients had systolic dysfunction, which is a predictor of outcome.	[27]	106
	Common sites of noncompaction were the apical, midinferior, and midlateral LV regions.	[28]	54
	The wall motion score index was markedly abnormal in the compacted LV segments and significantly less abnormal compared to the noncompacted segments.	[32]	17
	Noncompacted and compacted LV segments had comparable increased regional volumes and reduced systolic function: Systolic LV dysfunction was not confined to the noncompacted LV segments.	[32]	17
	All LV strains were decreased in all segments and LV-RS and LV-3DS showed further reduction in noncompacted segments compared to compacted segments.	[22] *	9
	Compared with controls, patients with preserved LV-EF had a larger mass, more abnormal LV geometry, and decreased global LV-LS.	[37]	17
	Global LV-LS was reduced regardless of LV-EF.	[41]	28
	Myocardial strain reduction was more significant in patients with focal myocardial fibrosis.	[45]	63
	Carriers of multiple genetic variants had a lower LV-EF and cardiac index, increased LV fibrosis, and reduced global LV-CS.	[54]	28
	Several strain abnormalities were seen even in children with LVNC.	[56]	16
	Certain strain parameters differed between LVNC and other cardiomyopathies.	[62]	35
	LV twist was reduced.	[72]	47
	There was a decreasing pattern in LV rotation values in patients’ relatives.	[53]	32 pts and 30 relatives
	There was no difference in the LV rotational pattern between dilated cardiomyopathy and LVNC.	[65]	42
	LV-RBR had high specificity and sensitivity in differentiating NCCM from LV hypertrabeculation.	[75]	52 pts with hypertrabeculation from which 34 proved to be NCCM
	Patients with LV-RBR showed worse NYHA functional status.	[73]	60
	Patients with lower LV-EF had higher rates of LV-RBR.	[47]	31
	Patients with clockwise LV-RBR had lower LV-LS but similar LV-EF.	[76]	28
	LV-RBR was present in 26–100% of patients and in most cases was oriented clockwise.	[74]	10
**Left atrium**	There were signs of LA remodeling.	[37]	17
	LA dimensions and volumes were larger.	[81]	320
	LA ejection force was increased.	[82]	17
	LA emptying fractions were smaller and certain peak global LA strains were reduced.	[20] *	12
	LA reservoir strain was an independent predictor of events.	[83]	95
	In the presence of a positive genotype, LA parameters were worse.	[54]	28
**Mitral valve**	MA was dilated and functionally impaired.	[87]	20
	The prevalence and grades of MV regurgitation were higher.	[88]	45
**Aortic valve**	Patients with bicuspid aortic valve did not harbour more LVNC and there was no evidence that they are at higher risk for the development of LVNC.	[93]	8, 49, 43
	Patients showed a higher ratio of AV regurgitation.	[88]	45
**Aorta**	The prevalence of ascending aortic dilation was low.	[96]	109
	Aortic stiffness was increased with no dilation but there was a reduced pulsatile change in diameter.	[97]	20
**RIGHT HEART**			
**Right ventricle**	RV apical trabecular thickness was greater.	[102]	105
	RV systolic dysfunction	[103]	56
	RV myocardium displayed more trabeculations.	[104]	20
	Subclinical impairment of RV myocardial deformation	[56]	16
**Right atrium**	RA volumes and certain stroke volumes were increased without RA strain abnormalities.	[19] *	13
**Tricuspid valve**	TA was dilated, with preserved sphincter-like function.	[18] *	21
	The prevalence and grade of TV regurgitation were higher.	[88]	45
**Pulmonary valve**	A low rate of patients showed pulmonary regurgitation.	[88]	45
**Pulmonary artery**	A high rate of patients had pulmonary hypertension.	[28]	54

Abbreviations: 3DS, three-dimensional strain; EF, ejection fraction; LA, left atrial; LS, longitudinal strain; LV, left ventricular; LVNC, left ventricular non-compaction; LV-RBR, left ventricular rigid body rotation; MV, mitral valve; pts, patients; RA, right atrial; RS, radial strain; RV, right ventricular; TV, tricuspid valve. The star (*) represents studies from the MAGYAR-Path Study. In some topics, results are contradictory.

## Data Availability

Not applicable.
